# Velocity and Pulsatility Measures in the Perforating Arteries of the Basal Ganglia at 3T MRI in Reference to 7T MRI

**DOI:** 10.3389/fnins.2021.665480

**Published:** 2021-04-26

**Authors:** Tine Arts, Timion A. Meijs, Heynric Grotenhuis, Michiel Voskuil, Jeroen Siero, Geert Jan Biessels, Jaco Zwanenburg

**Affiliations:** ^1^Department of Radiology, Center for Image Sciences, University Medical Center Utrecht, Utrecht, Netherlands; ^2^Department of Cardiology, University Medical Center Utrecht, Utrecht, Netherlands; ^3^Department of Pediatric Cardiology, University Medical Center Utrecht – Wilhelmina Children’s Hospital, Utrecht, Netherlands; ^4^Spinoza Center for Neuroimaging, Amsterdam, Netherlands; ^5^Department of Neurology, University Medical Center Utrecht, Utrecht, Netherlands

**Keywords:** flow velocity, perforating arteries, 3 tesla MRI, 7 tesla MRI, velocity pulsatility

## Abstract

Cerebral perforating artery flow velocity and pulsatility can be measured using 7 tesla (T) MRI. Enabling these flow metrics on more widely available 3T systems would make them more employable. It is currently unknown whether these measurements can be performed at 3T MRI due to the lower signal-to-noise ratio (SNR). Therefore, the aim of this study is to investigate if flow velocity and pulsatility in the perforating arteries of the basal ganglia (BG) can be measured at 3T MRI and assess the agreement with 7T MRI measurements as reference. Twenty-nine subjects were included, of which 14 patients with aortic coarctation [median age 29 years (21–72)] and 15 controls [median age 27 years (22–64)]. Using a cardiac-gated 2D phase-contrast MRI sequence BG perforating arteries were imaged at 3T and 7T MRI and perforating artery density (N_*density*_, #/cm^2^), flow velocity (V_*mean*_, cm/s) and pulsatility index (PI) were determined. Agreement between scanner modalities was assessed using correlation and difference plots with linear regression. A *p*-value ≤ 0.05 indicated statistical significance. It was shown that perforating artery flow velocity and pulsatility can be measured at 3T MRI (N_*density*_ = 0.21 ± 0.11; V_*mean*_ = 6.04 ± 1.27; PI = 0.49 ± 0.19), although values differed from 7T MRI measurements (N_*density*_ = 0.95 ± 0.21; V_*mean*_ = 3.89 ± 0.56; PI = 0.28 ± 0.08). The number of detected arteries was lower at 3T (5 ± 3) than 7T MRI (24 ± 6), indicating that 3T MRI is on average a factor 4.8 less sensitive to detect cerebral perforating arteries. Comparison with 7T MRI as reference showed some agreement in N_*density*_, but little to no agreement for V_*mean*_ and PI. Equalizing the modalities’ sensitivity by comparing the detected arteries on 7T MRI with the highest velocity with all vessels detected on 3T MRI, showed some improvement in agreement for PI, but not for V_*mean*_. This study shows that it is possible to measure cerebral perforating artery flow velocity and pulsatility at 3T MRI, although an approximately fivefold sample size is needed at 3T relative to 7T MRI for a given effect size, and the measurements should be performed with equal scanner field strength and protocol.

## Introduction

Cerebral perforating arteries are small branches of the main cerebral arteries that supply blood to the deep gray and white matter brain regions. Dysfunction of these perforating arteries plays a major role in cerebral small vessel disease (Wardlaw et al., 2013, 2019). These perforating arteries can be imaged using 7 tesla (T) magnetic resonance imaging (MRI) with a retrospectively gated 2D phase-contrast sequence (Guralnik et al., 1994; Bouvy et al., 2016; Geurts L. et al., 2018). Previous literature has shown that this sequence enables us to reliably measure the perforating artery flow metrics, i.e., blood-flow velocity and pulsatility (Bouvy et al., 2016). However, compared to 7T MRI scanners, 3T MRI scanners are more widely available in research institutions and hospitals. The ability to image the perforating arteries and measure their flow on 3T MRI would therefore considerably increase the employability of these metrics. However, the signal-to-noise ratio (SNR) of 3T MRI is lower than that of 7T MRI (Schenck et al., 2009), which means that 3T MRI will only be able to detect the relatively large perforating arteries with higher flow velocities. Therefore, in this study we investigate if flow velocity and pulsatility in the perforating arteries of the basal ganglia (BG) can be measured at 3T MRI and assess the agreement with 7T MRI measurements as reference. Also, we will explore whether the subset of perforating arteries detected at 7T MRI with the highest velocities are representative for the perforating arteries detected at 3T MRI. As a region of interest we chose the level of the basal ganglia (BG), as perforating arteries in this region are relatively large (up to 1 mm in diameter), which increases the likelihood of being detected with 3T MRI.

## Materials and Methods

### Image Acquisition

**FIGURE 1:**
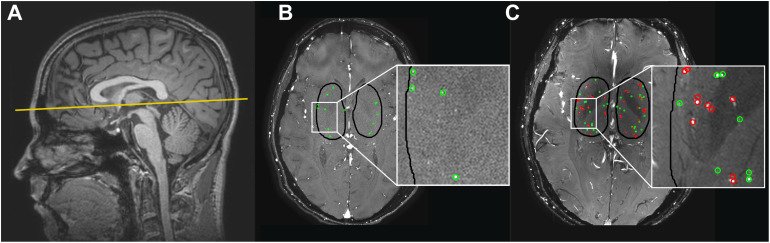
**(A)** The 2D phase contrast slice in the basal ganglia (BG) is located at the bottom of the corpus callosum (solid line). **(B,C)** Cerebral perforating arteries detected in the BG with 3 tesla (T) MRI **(B)** and 7T MRI **(C)** and included in the analysis are circled in green. Cerebral perforating arteries excluded from analysis due to a non-perpendicular orientation or close proximity to other perforating arteries (<1.2 mm) are circled in red. Note that for this subject all detected vessels at 3T were sufficient for inclusion. Also note the inhomogeneity in the background suppression at 7T (darker central area), which is induced by the well-known inhomogeneity of the radiofrequency B_1_^+^ transmit field (resulting in spatially varying flip angle) at 7T MRI (Van De Moortele et al., 2005).

Images for the technical comparison between 3T and 7T MRI were obtained from a larger, clinical study in which brain lesions and hemodynamics were studied in 14 patients with coarctation of the aorta (CoA) and 15 control subjects with no history of cardiovascular disease, neurological disease, or intellectual disability. The study was approved by our institutional review board. All subjects provided written informed consent. Patients with CoA were included when they had previously undergone surgical repair of CoA. Prior stent implantation for native or recurrent CoA was an exclusion criterion due to potential artifacts. General exclusion criteria comprised current pregnancy and the presence of a contraindication for undergoing MRI. Subjects were scanned on a Philips Ingenia Elition 3T scanner with a 20-channel Head-Neck-Spine coil and on a Philips Achieva 7T MRI scanner using a 32-channel receive head coil (Nova Medical, Wilmington, NC, United States; Philips Healthcare, Best, Netherlands). For the controls, 3T and 7T scanning sessions were performed on the same day, in random order. Patients’ 3T and 7T MRI scans were often performed on different days (*n* = 10, ranging from 96 to 227 days apart). On 7T MRI, small cerebral perforating arteries in the BG were imaged using a previously described retrospectively gated 2D phase-contrast (PC) sequence (Geurts L. et al., 2018). The 3T MRI 2D PC protocol was in principle the same as the 7T protocol, both with an acquired in-plane resolution of 0.3 mm × 0.3 mm, reconstructed to 0.2 mm × 0.2 mm in-plane resolution. Scan parameters of the 3T and 7T PC scans can be found in Table 1. A peripheral pulse oximeter was used for retrospective gating and cushions were placed besides the subject’s head to minimize head movement during scanning. The scan was planned manually, with a target slice location at the bottom of the corpus callosum (see Figure 1A). At 3T MRI, scanning was performed by the clinical technician on duty, and was therefore not always the same scanner operator for each subject. At 7T MRI, scanning was performed by the same scanner operator.

**TABLE 1:** Imaging parameters of the 2D phase contrast sequence of 3 tesla and 7 tesla MRI.

	3 tesla/7 tesla
FOV, mm (RLxAP)	250 × 250/180^*a*^
SENSE factor (AP direction)	1.5/1^*a*^
Acquired voxel size, mm^*b*^	0.3 × 0.3 × 2.0
Flip angle, °	50
Venc, cm/s	20
TR, ms	28.2/28
TE, ms	14.5/14.7–15.1
TFE factor	3/2^*c*^
Acquired temporal resolution (ms)	169/112
Reconstructed time points	9–10/13
Scan time, min:s^*d*^	3:27/5:28

*FOV, field of view; RL, right-left, AP, anterior-posterior; Venc, encoded velocity; TR, repetition time; TE, echo time; TFE, turbo field echo. ^*a*^The acquired matrix (FOV/voxel size/SENSE factor), which determines the scan time and affects the signal-to-noise ratio, is practically equal between 3T and 7T. ^*b*^Reconstructed to 0.2 mm × 0.2 mm in-plane resolution. ^*c*^The TFE factor determines the acquired temporal resolution (which is 2^∗^TFE^∗^TR); the 3T implementation didn’t allow for a lower TFE factor than 3. ^*d*^Scan time for a heart rate of 52 bpm.*

### Image Processing

2D PC scans were visually checked for sufficient quality, which meant that severe artifacts due to motion or large pulsating vessels should be absent. Scans with poor image quality were excluded from analysis.

The region of interest (ROI) was manually drawn around the BG (see Figure 1), using the magnitude 2D PC image. The anterior zone of this ROI was demarcated by the anterior end of the insula, the posterior zone by the posterior end of the thalamus, and the lateral zone by the claustrum (Bouvy et al., 2016). Small cerebral perforators in the BG were detected as previously published using Matlab (Geurts L. et al., 2018) (R2016b, The MathWorks, Natick, MA, United States), which includes the following steps: First, a phase correction was applied on the background to make the mean velocity of tissue 0 cm/s by median filtering the time-averaged velocity map and subtracting it from the velocity map of each cardiac time point. Then, the velocity SNR was calculated from an estimation of the magnitude SNR. Further, given the velocity SNR, the two-sided 95% velocity confidence intervals (CIs) were estimated for the mean blood flow velocity (V_*mean*_) to enable consistent selection of vessels with significant positive flow (i.e., non-zero, positive V_*mean*_ with a statistical significance of 0.05). All voxels inside the BG mask without 0 cm/s within their CI of V_*mean*_ and hyperintense signal in the magnitude image were considered significant. Only positive velocities with hyperintense magnitude signal were included to ensure that veins were not included in the selected vessels. Every group of neighboring significant voxels was defined as belonging to the same perforating artery and the voxel with the highest mean velocity of such a group was taken as representative for the perforating artery and used to calculate the flow measures. Perforating arteries in the BG with a non-perpendicular orientation to the scanning plane were excluded. This was performed by taking the perforating artery voxel and assessing the shape of the collection of surrounding high-intensity voxels. If the ratio of the largest axis length to the smallest axis length was more than a set threshold (2 in our case), the shape was determined to be elliptical and the small perforating artery was excluded. In addition, perforating arteries within 1.2 mm distance from each other were excluded from analysis as these are mostly multiple detections located on larger and/or non-perpendicular vessels. In Figure 1 the slice orientation and delineation of the BG as well as detected perforating arteries at 3T and 7T MRI are shown.

### Quantitative Measures

Analysis of the 3T as well as the 7T PC data resulted in a perforating artery density (number of detected perforators/cm^2^, N_*density*_). For each detected perforator, an average blood-flow velocity (V_*mean*_, cm/s), was obtained. The mean blood-flow velocity curve per subject was determined by averaging over all perforators. To calculate the pulsatility index (PI), the perforating arteries’ velocity curves were first normalized and then averaged. PI was calculated from the resulting mean normalized velocity curve with the following formula

(1) $$\text{PI}=\frac{{\text{V}}_{\max}-{\text{V}}_{\min}}{{\text{V}}_{\text{mean}}}$$

where V_*min*_, V_*max*_, and V_*mean*_, respectively, are the minimum, maximum, and mean of the mean normalized velocity curve (due to the normalization procedure V_*mean*_ equals 1.0).

### Statistical Analysis

The agreement between N_*density*_, V_*mean*_, and PI at 7T and 3T MRI was quantified and visually shown using correlation plots combined with linear regression as well as difference plots combined with linear regression (Martin Bland, 1986; Pollock et al., 1992; Giavarina, 2015). The next sections briefly provide background information on correlation plots and difference plots, after which the details of the methods to compare the 3T and 7T results are described.

#### Correlation Plots

The correlation plots combined with linear regression determine the coefficient of determination (*r*^2^), the latter with an associated *p*-value, providing information regarding the coefficient’s significance. In case of significance, the coefficient of determination shows the amount of shared inter-subject variance between 7T MRI and 3T MRI results. If significance is absent, shared inter-subject variance is lost in the noise.

#### Difference Plots

Difference plots in absolute units (i.e., Bland Altman plots or absolute difference plots) (Martin Bland, 1986; Bland and Altman, 1995) provide information about how the inter-method variance compares to the inter-subject variance. A large inter-method variance compared to the mean values suggests that the measurement noise dominates over the inter-subject variance, resulting in limited agreement between the methods. However, a small inter-method variance compared to the mean values implies a high level of agreement between the two methods. By using Bland Altman plots with linear regression and a resulting *p*-value, a possible significant proportional bias (*p*-value ≤ 0.05) can be studied between the differences and the measured values. Proportional bias indicates that the difference of the measured values of the two modalities scales with the average of the two values, i.e., the differences are (partly) predicted by the mean values. In case of a significant proportional bias, the bias is further investigated to assess its characteristics. To this end, the difference plots are presented in percentage unit differences (i.e., Pollock plots or relative difference plots) (Pollock et al., 1992) and likewise combined with linear regression. If significant proportional bias exists in the absolute difference plots, but not in the relative difference plots, it can be concluded that the bias between 3T and 7T MRI is mainly linear. However, if significant proportional bias is present in the absolute difference plots as well as in the relative difference plots, also considerable non-linear sources of error are present. Measurement error is therefore not constant in an absolute or relative sense, strongly limiting the agreement (Giavarina, 2015).

#### 3T-7T Comparison Using Correlation and Difference Plots

First, we determined whether significant differences existed between patients and controls in N_*density*_, V_*mean*_, and PI at 7T MRI using an unpaired Student’s *t*-test. A *p*-value ≤0.05 was considered statistically significant. Significant differences at 7T MRI would allow us to further evaluate the 3T MRI for its ability to detect a certain effect size. Further, correlation and difference plots were constructed for all detected perforating arteries on 3T and 7T MRI, and linear regression was applied. A secondary analysis was performed in which the bias caused by the higher sensitivity of the 7T MRI was minimized. This was done with two matching approaches: (i) matching by number, in which the number of analyzed vessels was made equal per subject between 3T and 7T MRI by selecting the *N* vessels at 7T with the highest velocities, with *N* the number of vessels detected at 3T MRI and (ii) matching by location, by visually matching 3T and 7T MRI vessels on anatomical location. For both approaches correlation and difference plots were constructed. However, concerning matching by location, 2D PC planning of 3T and 7T MRI was sufficiently similar for matching in only 11 of 28 subjects. In these 11 subjects, a total of 17 matched pairs were found (range 1–4 per subject). This limited number of suitable subjects and matches resulted in limited power. Therefore, in this study, vessel matching by anatomical location was not seen as a suitable approach to compare small vessel flow measures across two scanning modalities. The results of this approach are shown in Supplementary Material A.

Statistical analyses were performed in IBM SPSS (IBM Corp., v22.0, NY, United States) and GraphPad Prism (GraphPad Software, v5.03, CA, United States).

## Results

The 3T 2D PC scan of one patient exhibited excessive motion artifacts, therefore this subject was excluded from analyses. Data of the remaining 13 patients [median age 29 years (range 21–72 years)] and 15 controls [median age 27 years (range 22–64 years)] were included.

At 3T as well as at 7T MRI, cerebral perforating arteries were detected for each subject. At 7T MRI, perforating artery measurements of patients did not significantly differ from those of controls (see Supplementary Material B). As group differences in perforating artery measures could therefore not be used to compare 3T and 7T MRI, the subject groups were pooled for the remaining analyses.

The number of detected perforating arteries (N_*detected*_) at 3T MRI and 7T MRI was (mean ± standard deviation) 5 ± 3 and 24 ± 6, respectively.

### Agreement Between 7T and 3T MRI Perforating Artery Flow Measures, Using All Detected Perforating Arteries

**FIGURE 2:**
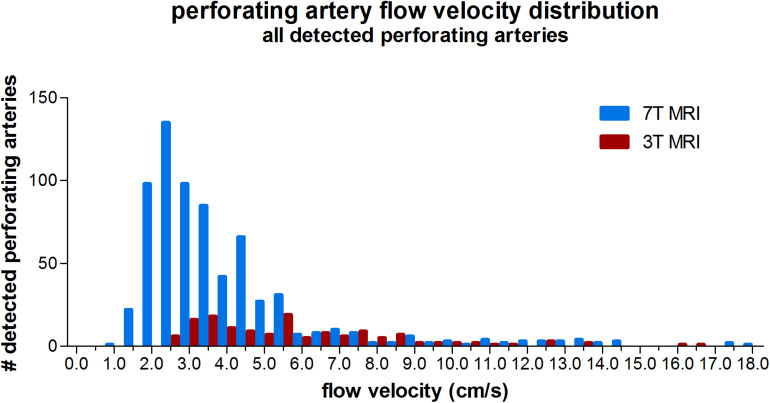
Histogram showing the velocity distribution of all detected perforating arteries on 3 tesla and 7 tesla MRI of all subjects.

Three tesla MRI detected less perforating arteries with low velocities compared to 7T MRI, as can be seen from the velocity distribution in Figure 2. For higher velocities, the number of detected perforating arteries became more similar between 3T and 7T MRI for a given velocity.

**FIGURE 3:**
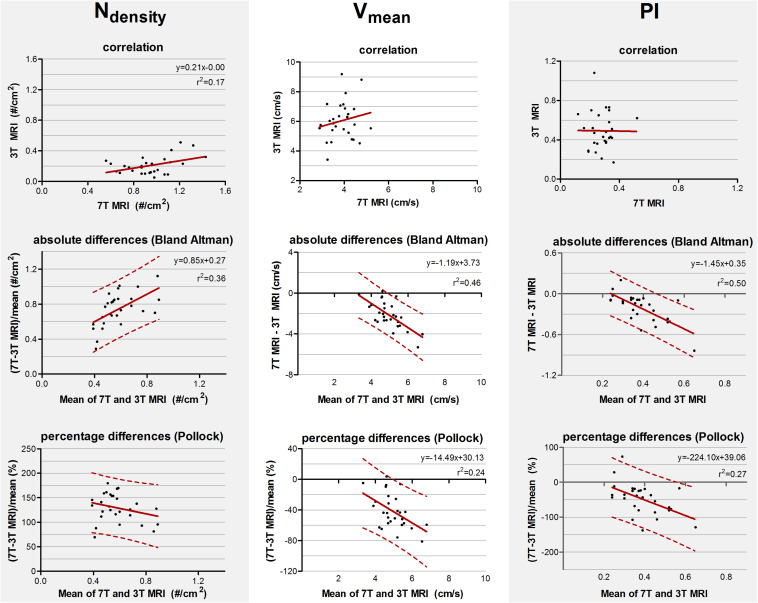
Top row: Correlation plots of the number of detected perforating arteries/cm^2^ (N_*density*_), the mean blood flow velocity (V_*mean*_) and pulsatility (PI) measured at 3 tesla (T) and 7T MRI. The line resulting from linear regression is given by the solid line. In case of a significant correlation the equation of the regression line is given. Middle and bottom row: difference plots in absolute units (Bland Altman) and in percentage units (Pollock), respectively, for N_*density*_, V_*mean*_, and PI. The regression line (i.e., bias) (solid line) and the limits of agreement (dotted lines) are shown. In case of a significant linear regression, the equation of the regression line is presented.

The mean and standard deviations of N_*density*_, V_*mean*_, and PI are given in Table 2. The correlation and absolute and relative difference plots for these variables are shown in Figure 3. For N_*density*_, the coefficient of determination *r*^2^ of the correlation showed a weak but significant correlation (*r*^2^ = 0.17, *p* = 0.02). For V_*mean*_ (*p* = 0.34) and PI (*p* = 0.97) no significant coefficient of determination was seen in the correlation plots.

**TABLE 2:** Perforating artery flow at 3 and 7 tesla MRI using all detected perforating arteries.

	3 tesla (*n* = 28)	7 tesla (*n* = 29)
N_*density*_ (#/cm^2^)	0.21 ± 0.11	0.95 ± 0.21
V_*mean*_ (cm/s)	6.04 ± 1.27	3.89 ± 0.56
PI	0.49 ± 0.19	0.28 ± 0.08

*Results are presented as mean ± standard deviation. N_*density*_, number of detected perforating arteries/cm^2^; V_*mean*_, mean blood-flow velocity; PI, pulsatility index.*

For N_*density*_, V_*mean*_, and PI, the inter-method variances were equal or even larger compared to the average values (see Bland Altman plots, Figure 3). For N_*density*_, linear regression of the Bland Altman plots showed a significant linear bias (*r*^2^ = 0.36, *p* = 0.001) indicating that the difference between the measured values scales with the average of the measured values. This was also true for V_*mean*_ (*r*^2^ = 0.46, *p* < 0.001) and PI (*r*^2^ = 0.50, *p* < 0.001).

For N_*density*_, linear regression of the relative differences against the mean no longer showed a significant proportionality of the bias (*p* = 0.16) indicating that the earlier detected dependency of the absolute differences on the measured value, is mainly linear bias. However, V_*mean*_ and PI still showed a significant proportional bias in the relative difference plots (*r*^2^ = 0.24, *p* = 0.01 and (*r*^2^ = 0.27, *p* = 0.005, respectively).

### Agreement Between 3T and 7T MRI Perforating Artery Flow Measures in Case of Matched Perforating Artery Detection Sensitivity

**FIGURE 4:**
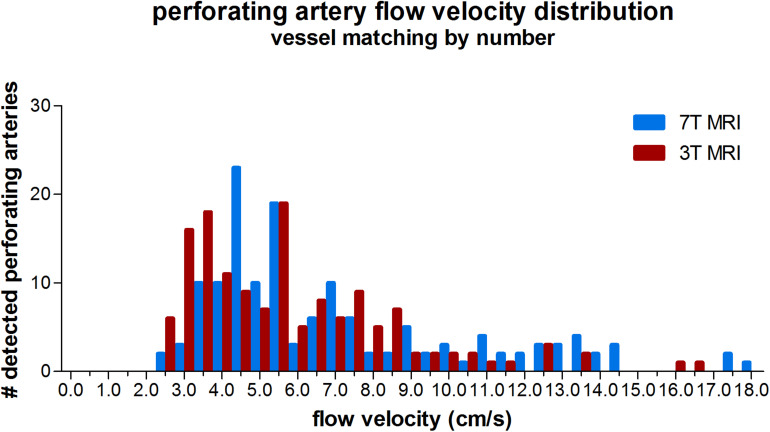
Histogram showing the velocity distribution of all detected vessels of 3 tesla (T) MRI vs. 7T MRI, where for 7T for each individual only the *N* vessels with highest velocities were taken (*N* refers to the number of vessels detected on 3T MRI for a given subject), in order to equalize the sensitivity to detect cerebral perforating arteries for both modalities.

The velocity distribution of all detected perforating arteries on 3T MRI and the detected perforating arteries with highest velocities on 7T MRI (matching the number of 7T arteries to the number detected on 3T to avoid the bias induced by the higher sensitivity on 7T MRI) is shown in Figure 4. A more equal velocity distribution of the perforating arteries between 7T and 3T MRI can be seen. However, a paired Student’s *t*-test still showed a significant difference between the two scanning modalities in V_*mean*_ (*p* < 0.001) and PI (*p* = 0.03).

**FIGURE 5:**
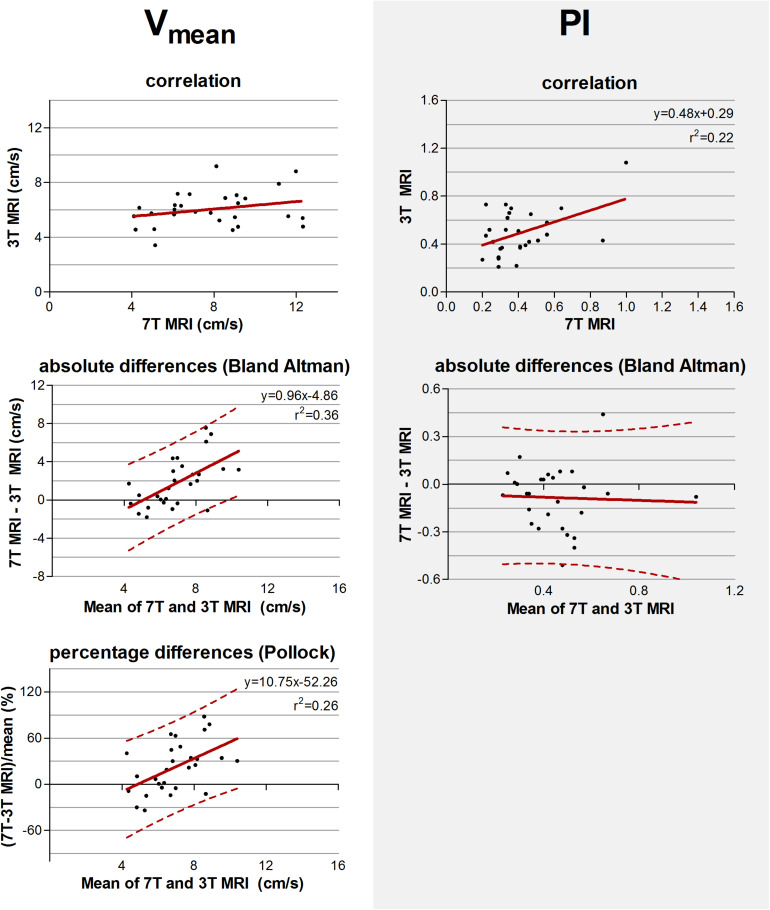
Top row: Correlation plots of the mean blood flow velocity (V_*mean*_) and pulsatility index (PI) determined of all detected perforating arteries at 3 tesla (T) and of the detected perforating arteries with highest velocities at 7T MRI. The line resulting from linear regression is given by the solid line. In case of a significant correlation the equation of the regression line is given. Middle and bottom row: difference plots in absolute units (Bland Altman) and in percentage units (Pollock), respectively, for V_*mean*_ and PI. The regression line (i.e., bias) (solid line) and the limits of agreement (dotted lines) are shown. In case of a significant linear correlation, the equation of the regression line is presented. If proportional bias is absent in the Bland Altman plot, no Pollock plot is shown.

The measured values and standard deviations of V_*mean*_ and PI, of 7T and 3T MRI are given in Table 3. The correlation and absolute and relative difference plots for these variables are shown in Figure 5. Linear regression revealed no significant correlation for V_*mean*_ (*p* = 0.17), contrary to PI, for which the coefficient of determination was significant (*r*^2^ = 0.22, *p* = 0.01), indicating that some of the variation in PI on 3T MRI was explained by variation of PI on 7T MRI.

**TABLE 3:** Perforating artery flow at 3 and 7 tesla MRI in case of vessel matching by number.

	3 tesla (*n* = 28)	7 tesla (*n* = 28)
V_*mean*_ (cm/s)	6.04 ± 1.27	7.85 ± 2.44
PI	0.49 ± 0.19	0.41 ± 0.18

*Results are presented as mean ± standard deviation. V_*mean*_, mean blood-flow velocity; PI, pulsatility index.*

For V_*mean*_, the Bland Altman plot shows large inter-method variance compared to inter-subject variance. For PI, however, the two variances were quite equal. Linear regression of the Bland Altman plots for V_*mean*_ showed a significant linear bias (linear regression in the Bland Altman plot; *r*^2^ = 0.36, *p <* 0.001). This bias was still present in the Pollock plots (*r*^2^ = 0.26, *p* = 0.01), indicating that the measurement error is not merely linear in an absolute or relative sense. PI showed no significant linear bias (linear regression in the Bland Altman plot; *p* = 0.84).

## Discussion

This study shows that perforating artery flow velocity and pulsatility can be measured at 3T MRI at the level of the BG, though the values found on 3T MRI differed from those obtained at 7T MRI. Comparison of all detected vessels on 7T and 3T MRI suggests agreement in N_*density*_, but little to no agreement for V_*mean*_ and PI. When equalizing the modalities’ sensitivity by comparing the detected vessels on 7T MRI with the highest velocities with all vessels detected on 3T MRI, we find an improved agreement for PI, but not for V_*mean*_. From the fact that the standard deviations of V_*mean*_ and PI are about twice as large at 3T as at 7T MRI, while only one-fifth of the number of vessels is detected per cm^2^ (Table 2 vs Table 3), we conclude that the measurement noise strongly dominates any physiological inter-subject differences and temporal intra-subject variability in the current measurements: the contribution of both physiological inter-subject differences and intra-subject temporal variability to these standard deviations would not scale with the number of detected vessels. Large group sizes are therefore required for sufficient power to detect differences in perforating artery conditions in, for example, disease. Below, we will discuss the observations in light of the differences in sensitivity between 3T and 7T MRI, 2D PC slice planning differences, and in light of the known measurement errors that exist due to the fact that these perforators have diameters typically smaller than the voxel size of the measurements. These measurement errors depend on 3T and 7T MRI specific details, and thus vary with field strength, which further limits the agreement. It is therefore important to be thoughtful regarding the study design, and to use an equal MRI scanner strength and protocol in future clinical studies.

### Sensitivity

**FIGURE 6:**
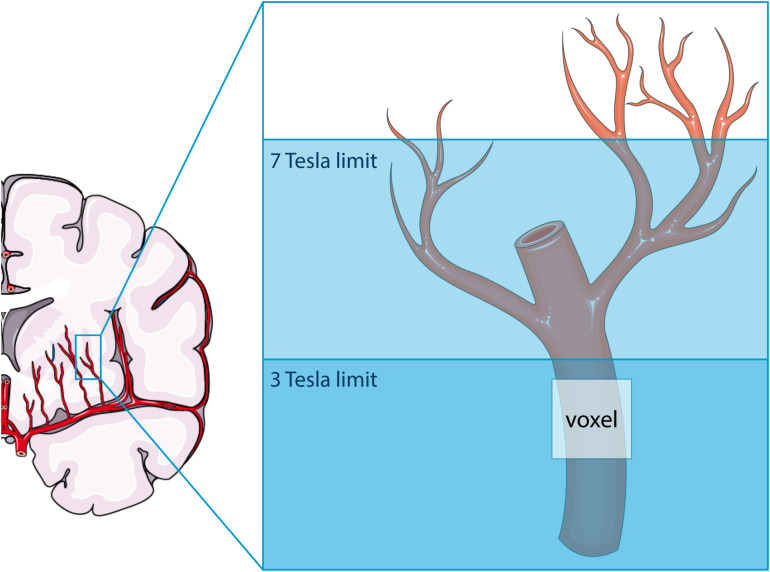
Schematic representation of the difference in sensitivity between 3 tesla (T) and 7T MRI for assessing flow and pulsatility in the perforating arteries in the basal ganglia. Both modalities can only detect the proverbial tip of the iceberg, albeit that this tip is larger at 7T than at 3T MRI. The voxel shown indicates the presence of partial volume effects, which are thus different between 3T and 7T MRI due to the difference in the proverbial tip of the iceberg. This image was created using Servier Medical Art templates, which are licensed under a Creative Commons Attribution 3.0 Unported License; https://smart.servier.com.

As illustrated in Figure 6, the higher SNR at 7T MRI makes the 2D PC at 7T sensitive to smaller, more downstream vessel segments compared to 3T. This has two consequences: (a) more detected vessels at 7T MRI and (b) lower velocities at 7T, since the additional vessels at 7T are smaller vessels. Both consequences were recognized when all detected vessels were included in the analysis. The tendency of 7T MRI to reflect on average smaller vessels also explains the negative trend in the difference plot of V_*mean*_ when all vessels are included. Whereas the 7T mean velocity is relatively stable due to a high prevalence of small vessels with low velocity (see Figure 2), the 3T mean velocity is quickly dominated by a few relatively large vessels (velocity “outliers” in the relatively flat histogram), which explains why an average higher velocity is accompanied by a higher difference in V_*mean*_ between 3T and 7T. Similar behavior as for V_*mean*_ is seen for the PI as well, as larger vessels also have larger pulsatility.

### Contrast Differences Due to Partial Volume Effects

Since the diameters of the majority of the detected perforating arteries are lower than the voxel size, partial volume effects cause non-linear errors in the measured velocities and the derived pulsatilities. Partial volume effects lead to an underestimation of the velocity and an overestimation of the pulsatility, as has been described and quantified by simulations in previous work (Bouvy et al., 2016; Geurts L.J. et al., 2018). Since we used the same acquired resolution for 3T and 7T MRI, the 7T measurements are more affected by partial volume effects due to a larger vessel diameter-voxel size mismatch (see Figure 6), as they include more distal/smaller vessels. This dissimilarity can explain the non-linear behavior of the differences between 3T and 7T as reflected by the relative difference (Pollock) plots when all vessels were included in the analysis. One could expect that the influence of partial volume effects would be minimized in the secondary analysis, where we discarded the vessels with the lowest velocities from the 7T measurements. By doing this, we focused on a similar segment of the vascular tree and, thus, to similar velocities and partial volume fractions. However, partial volume effects on the velocity not only depend on the volume fraction in the voxel that is occupied by the vessel, but also on tissue and imaging parameters, such as the longitudinal relaxation time T_1_ and the flip angle. At 7T, the tissue T1 values are generally longer, which leads to better background suppression for a given flip angle, and, thus to less velocity underestimation compared to a similar vessel and partial volume fraction at 3T. The velocity histogram for the analysis with matched vessel detection sensitivity and thus matched vessel numbers indicates that the partial volume effect indeed appears to be worse at 3T, as we see more vessels with low velocities (<4 cm/s) at 3T. This explains the positive trend in the difference plot for V_*mean*_ for the analysis with matched detection sensitivity, as higher mean velocities on the x-axis of the Bland–Altman plot now are mainly due to higher 7T velocities, and cause, thus, a positive difference between the 7T and 3T MRI measurements. The fact that the PI no longer shows a linear bias in the difference plot for the analysis with matched vessel detection sensitivity, suggests that this analysis indeed effectively focuses on a similar segment of the vascular tree and that the remaining underestimation of the velocities due to the partial volume effects drops out in the calculation of PI.

The values found in this study for N_*detected*_, V_*mean*_, and PI at 7T MRI are lower than those found in earlier studies (Bouvy et al., 2016; Geurts L. et al., 2018). This may be attributable to the exclusion of non-perpendicular perforating arteries in the basal ganglia, which are often larger perforating arteries with higher velocities and pulsatilities. Also, the mean age of our subject group is lower, which could result in lower PI values (Zarrinkoob et al., 2016).

### Consequences of the Field Strength Dependence

The results presented in this paper show that the measurements of perforator velocity and pulsatility are strongly field strength dependent. This does not mean that impaired flow or pulsatility of the microvessels cannot be detected using 3T MRI but does show the importance of acquiring data on scanners of equal field strength and with equal scanning parameters when comparing subject cohorts.

The fact that measurement noise strongly dominates any natural inter-subject differences and temporal physiological variations, together with the observation that on 3T MRI almost one-fifth of the number of vessels is detected per cm^2^ compared to 7T MRI, implies that at 3T MRI one would need a subject cohort almost five times larger as needed at 7T MRI to detect a given effect size. However, this is only true under the assumption that the perforating arteries detected on 3T MRI are physiologically similar to those detected on 7T MRI. To the best of our knowledge, it is currently unknown to what extent cerebral small vessel disease affects the larger perforating arteries as visible at 3T in a different way than the smaller perforating artery segments to which 7T MRI is more sensitive.

### Limitations

This study also has a few limitations. First, planning of the 2D PC slice was done manually, on 3T as well as 7T MRI, by different scanner operators. This may have caused slight differences in the slice location. This, together with physiological intra-subject variation and subject repositioning, was the main reason why vessel matching by location of the 3T and 7T acquisition was feasible in only a limited number of subjects, yielding only a total of 17 matched vessels. In addition, a direct vessel-to-vessel comparison does not allow to average over vessels. As a result, measurement noise remains in the flow measures, which further complicates vessel-to-vessel flow comparison. This is particularly true for the pulsatility index as discussed in more depth before (Arts et al., 2021). Therefore, in this study, vessel matching by anatomical location was not seen as a suitable approach to compare small vessel flow measures across two scanning modalities. Nonetheless, differences in the agreement between the approach to match vessels by number (using scans with similar and deviant 2D PC planning) and by location (using only scans with similar 3T and 7T planning) show that planning dissimilarities contribute considerably to the agreement between the scanning modalities. Despite the fact that the 2D PC slice planning was protocolled, slice planning variation was still present. This was particularly true at 3T MRI, where the planning was performed by different clinical technicians. Therefore, the MRI data acquisition as performed in this study is representative for daily clinical practice.

A second limitation concerns the lower temporal resolution of the 3T MRI protocol compared to the 7T MRI protocol. As the 3T MRI study was integrated in the clinical workflow, it required the use of the vendor supplied scanner control software prohibiting the use of higher temporal resolutions (minimum TFE factor of 3, see Table 1). Lower temporal resolution leads to flattening of the velocity curve, and thus to underestimation of the PI. Even at 7T, the temporal resolution was limited (Geurts L. et al., 2018). It seems that a further reduction of the temporal resolution in the current 3T protocol had limited effect on PI, as 3T MRI PI values were not systematically lower than the 7T MRI PI values for the matched analysis (Figure 5).

Third, as performed on 7T MRI in previous literature (Geurts L. et al., 2018), 3T MRI 2D PC repeatability studies should be completed. Knowing the uncertainty from repeated measurements of the velocity and pulsatility, will allow calculation of the sample sizes needed for future clinical studies. Given the fact that the measurement noise apparently dominates inter-subject variance in the current 3T MRI results as argued above, the reported inter-subject standard deviation of Table 2 may serve as preliminary uncertainty metrics for power calculations.

A final limitation is that no significant differences were present between patients and control subjects at 7T MRI, limiting the dynamical range of our subject cohort. However, pooling these subject groups did increase our sample size.

A strength of this study is the fact that the majority of included subjects was scanned on the same day, with the 3T and 7T MRI performed close together in time. This minimalizes the intra-subject variability. Also, the 3T and 7T scanning sessions were performed in random order. This diminishes bias due to, for example, more subject nervousness and accompanying motion during the first scanning session compared to the second scanning session.

## Conclusion

In this study it was shown that cerebral perforating artery velocity and pulsatility measurements can be performed at 3T MRI, paving the way for more microvascular flow research in relation to cerebrovascular disease. Although 3T MRI shows little agreement with 7T MRI concerning the perforating artery flow measurements, these differences can be explained from the fact that both field strengths can measure only the proverbial tip of the iceberg of the perforating arteries, resulting in scanner-dependent biases. Consequently, future clinical studies should use equal scanning protocol and field strength for all participants. The lower sensitivity of 3T MRI to detect perforating arteries warrants the use of larger cohorts for detecting a given effect size, compared to a similar study at 7T. The drawback of the larger sample sizes may however be compensated by the much larger availability and lower scanning costs of 3T MRI.

## Data Availability Statement

The raw data supporting the conclusions of this article will be made available by the authors, without undue reservation.

## Ethics Statement

The studies involving human participants were reviewed and approved by the Ethical Review board of the University Medical Center Utrecht. The patients/participants provided their written informed consent to participate in this study.

## Author Contributions

TA, TM, JZ, and GB contributed to the conception and design of the study. HG, MV, TM, and JZ were responsible for data acquisition. TA was responsible for data analysis and wrote the first draft of the manuscript. TA, JS, and JZ were responsible for data interpretation. TM wrote sections of the manuscript. All authors contributed to critical revisions of the manuscript and approved the submitted version.

## Conflict of Interest

The authors declare that the research was conducted in the absence of any commercial or financial relationships that could be construed as a potential conflict of interest.
