# Supplementary material for: Velocity and Pulsatility Measures in the Perforating Arteries of the Basal Ganglia at 3T MRI in Reference to 7T MRI
Source: Front Neurosci. 2021 Apr 26;15:665480. doi: 10.3389/fnins.2021.665480 (PMC8107291; doi:10.3389/fnins.2021.665480)
Supplement: Supplementary file 1 [file Table_1.docx]

Supplementary Material A

In Table A1 the measured values and standard deviations of the mean blood flow velocity (V_mean_) and the pulsatility index (PI), of 7 tesla (T) and 3T MRI, are given, after visually matching vessels by anatomical location. A paired Student’s t-test showed no significant difference between the two scanning modalities in V_mean_ (*p*=0.49) or PI (*p*=0.17).

The correlation and absolute difference plots for these variables are shown in Figure A1. For V_mean_ (*p*=0.07) as well as PI (*p*=0.26) no significant coefficient of determination was seen in the correlation plots. Bland Altman plots show that for V_mean_ and PI the inter-method variances were larger than the average values (see Bland Altman plots), implying limited agreement between modalities. However, for V_mean_ and PI the Bland Altman plots showed no significant linear bias, indicating that the difference between the measures values does not scale with the average of the measured values.

Results of this approach, i.e. visually matching vessels by anatomical location, suggest more agreement compared to the approach of matching vessels by number. Unlike for matching by number, no differences were seen in V_mean_ and PI between field strengths, nor linear biases in the Bland-Altman plots. This is likely due to two reasons. First, matching by location compares the same vessel across scanning modalities, making the vessel size equal at 3T and 7T MRI. Therefore, partial volume differences can only result from T1 differences, and not from partial volume fraction. In fact, the impact of T1 differences even appears to be small, since it would result in more velocity underestimation at 3T MRI compared to 7T MRI which is not evident in Table A1. Second, vessel matching can only be achieved in case of sufficiently similar planning of the 2D phase contrast (PC) slice at 3T and 7T MRI. So, differences between 3T and 7T MRI due to planning dissimilarities are absent.

However, an important remark should be made regarding the noise in these resulting measurements. This was firstly due to 2D PC planning differences, yielding only a total of 17 matched vessels in 11 out of 28 subject. Second, this direct vessel-to-vessel comparison resulted in noisy flow measures, particularly for PI as shown in previous research (1). Therefore, vessel matching by anatomical location was not seen as a highly suitable approach to compare small vessel flow measures across two scanning modalities.

| Table A1: Perforating artery flow at 3 and 7 tesla MRI in case of vessel matching by location | | |
| --- | --- | --- |
|  | **3 tesla (n=28)** | **7 tesla (n=28)** |
| V_mean_ (cm/s) | 4.97±2.91 | 4.72±2.91 |
| PI | 0.77±0.30 | 0.92±0.40 |
| Results are presented as mean±standard deviation. Abbreviations: V_mean_= mean blood-flow velocity; PI= pulsatility index. | | |

| 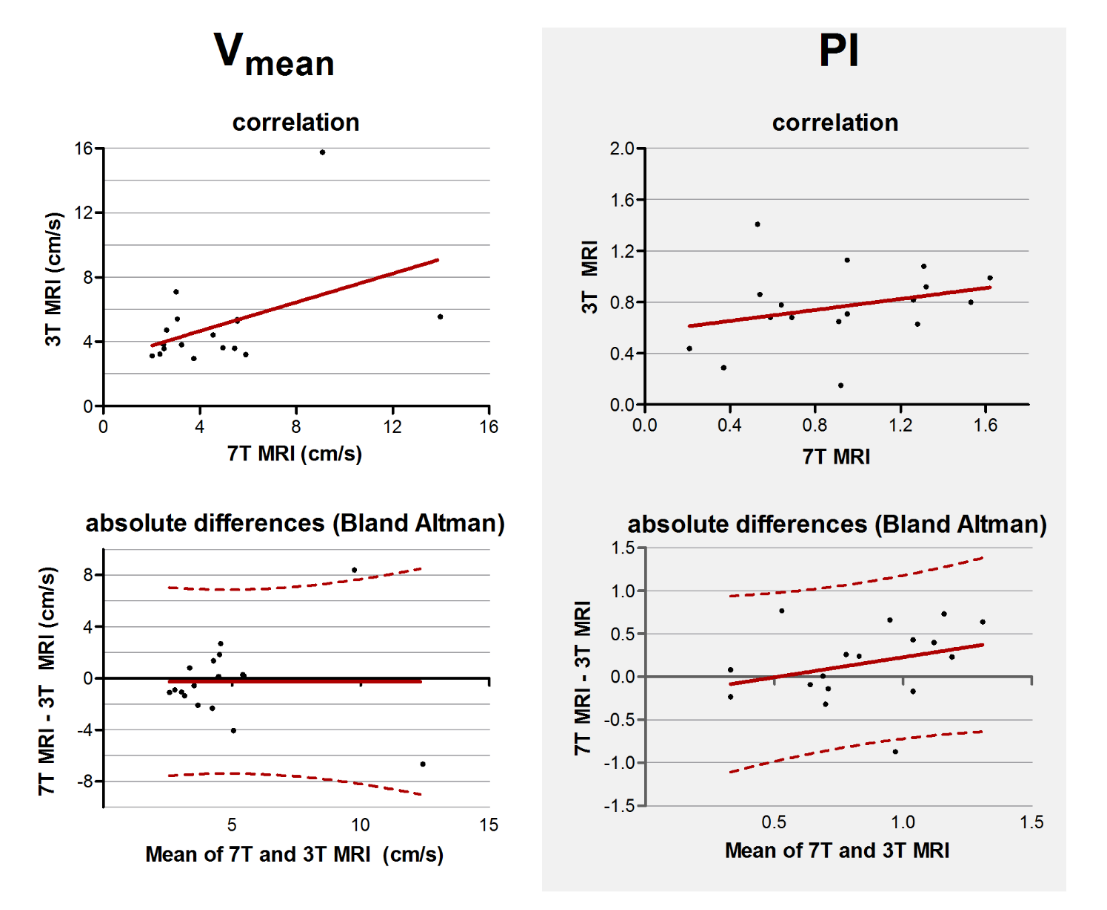 |
| --- |
| Figure A1: Top row: Correlation plots of the mean blood flow velocity (V_mean_) and pulsatility index (PI) measured at 3 tesla (T) and 7T MRI. The line resulting from linear regression is given by the solid line. Middle and bottom row: difference plots in absolute units (Bland Altman) for V_mean_ and PI. Because proportional bias is absent in the Bland Altman plots, no Pollock plots are shown. The regression line (i.e. bias) (solid line) and the limits of agreement (dotted lines) are shown. |

1. Arts T, Siero JCW, Biessels GJ, et al. Automated Assessment of Cerebral Arterial Perforator Function on 7T MRI. *J Magn Reson Imaging*. 2021;53:234-241. doi:10.1002/jmri.27304.

Supplementary Material B

| Table B1: Perforating artery flow measures at 7 tesla MRI of patients and controls | | | |
| --- | --- | --- | --- |
|  | **Patients** | **Controls** | ***p*-value** |
| N_density_ | 0.97±0.18 | 0.93±0.23 | 0.60 |
| V_mean_ | 3.91±0.59 | 3.87±0.54 | 0.85 |
| PI | 0.27±0.09 | 0.29±0.06 | 0.17 |
| Results are presented as mean±standard deviation. *P*-values are determined using an unpaired Student’s t-test and *p*≤0.05 was considered statistically significant. Abbreviations: N_density_= number of detected perforating arteries/cm^2^; V_mean_= mean blood-flow velocity; PI= pulsatility index. | | | |
